# A nano-graphite cold cathode for an energy-efficient cathodoluminescent light source

**DOI:** 10.3762/bjnano.4.58

**Published:** 2013-08-28

**Authors:** Alexander N Obraztsov, Victor I Kleshch, Elena A Smolnikova

**Affiliations:** 1 Department of Physics, M.V. Lomonosov Moscow State University, Moscow 119991, Russia; 2 Department of Physics and Mathematics, University of Eastern Finland, Joensuu 80101, Finland

**Keywords:** cathodoluminescence, electron field emission, light source, nano-graphite, vacuum electronics

## Abstract

The development of new types of light sources is necessary in order to meet the growing demands of consumers and to ensure an efficient use of energy. The cathodoluminescence process is still under-exploited for light generation because of the lack of cathodes suitable for the energy-efficient production of electron beams and appropriate phosphor materials. In this paper we propose a nano-graphite film material as a highly efficient cold cathode, which is able to produce high intensity electron beams without energy consumption. The nano-graphite film material was produced by using chemical vapor deposition techniques. Prototypes of cathodoluminescent lamp devices with a construction optimized for the usage of nano-graphite cold cathodes were developed, manufactured and tested. The results indicate prospective advantages of this type of lamp and the possibility to provide advanced power efficiency as well as enhanced spectral and other characteristics.

## Introduction

The fundamental importance of light in our lives cannot be overstated. The sun is the only natural source of light emission with appropriate intensity. This is the driving force for the elaboration of artificial light sources. The demand on artificial lighting increases constantly and will continue to increase in the future. The conversion of electric energy is the most practical way for light generation and it is currently used in incandescent bulbs, gas discharge, and electroluminescent lamps of various designs, shapes, input and output power. Additionally, a photoluminescent process is used to convert blue or ultraviolet radiation, produced by gas discharge or by electroluminescence, to white light. Unfortunately, because of the fundamental principles of nature, the energy efficient generation of light requires the usage of extremely toxic materials (mercury, heavy metals and others). This leads to the necessity of expensive and laborious efforts to dispose of the mercury-based fluorescent devices and the semiconductor-based light emitting diode (LED) lamps (see, e.g., [1–2]). Moreover, the spectral characteristics of the light produced by these fluorescent and LED lamps are often not perceived as pleasing in contrast to incandescent lamps. But incandescent bulbs convert only 5% of the consumed energy into light and are thus considered as ineffective. The other 95% of the energy are transformed into heat, which cannot be considered as waste in many countries where electricity is used for house heating practically every day. In the “energy efficient” fluorescent and LED lamps the energy conversion ratio is about 10%, so that there is a decrease of energy loss on heating only from 95 to 90%. At the same time, production costs for these lamps, i.e. consumption and waste of energy at the production plant, are many times higher compared to the production costs of incandescent bulbs.

Thus, the development of new types of light sources is necessary to provide better energy efficiency, spectral characteristics, and other properties desired by the consumer. The process of cathodoluminescence (CL), which is potentially able to provide a conversion of up to 35% [3] (or more for nanostructured phosphors [4]) of the energy of the excited electron into radiation, is therefore attractive for light generation [5]. The most suitable source of electrons is the field emission (FE) cathode [5], allowing to exploit the FE effect for the creation of CL light emitting lamps. Cathodes of this type (also called "cold cathodes") are capable of generating intense electron beams virtually without any energy consumption because of the quantum tunneling nature of the FE effect [6]. Individual field emitters are required to have a needle- or blade-shape with a high aspect ratio in order to provide a sufficient intensity of the electric field at moderate voltages. Multi-emitter cathodes are necessary to achieve a reasonable total intensity of electron beams, because the current from a single emitter is limited due to its small emission surface area. To prevent field shielding the individual emitters, composing flat multi-emitter FE cathodes, must be separated from each other by a distance a few times larger than the height of the emitters [7–8]. To survive under the action of the extremely strong electric field, FE cathodes must be made from rather strong materials – hard metals, or selected semiconductors. From this point of view graphite-like materials, having the strongest interatomic interaction, are attractive for the FE cathode production. In this paper we describe the production technique and the electron field emission (FE) characteristics of nano-graphite films (NGF) and prototypes of CL lamps with NGF cold cathodes.

## Results and Discussion

**Figure 1:**
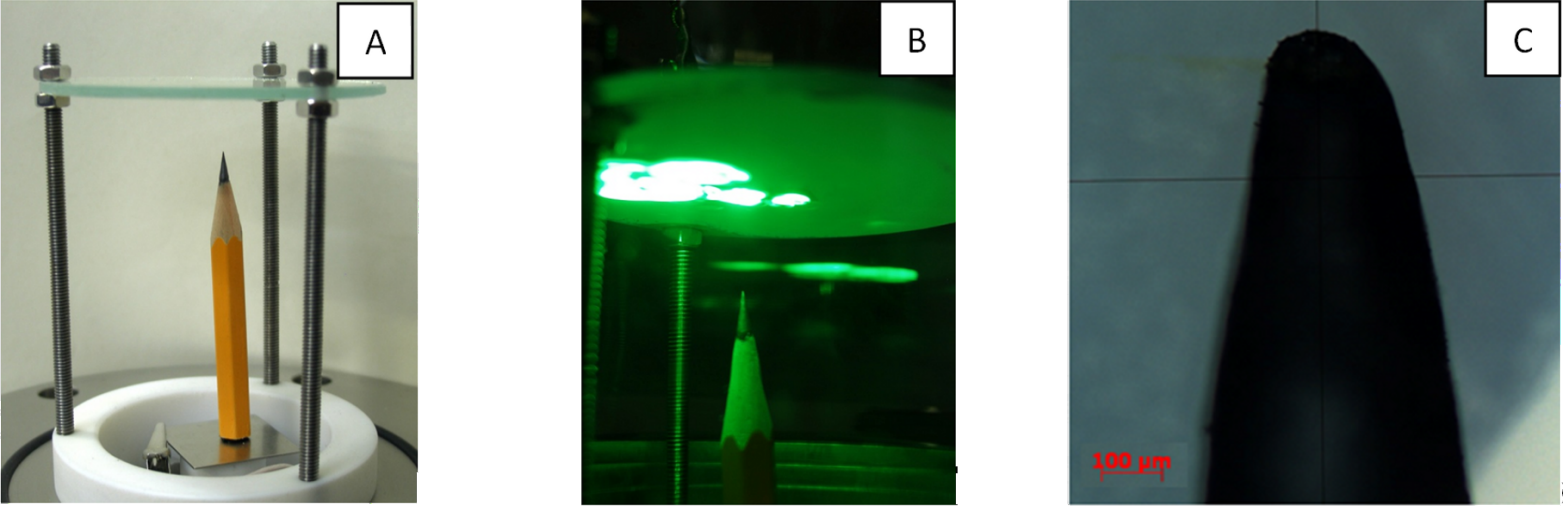
Experimental setup with a pencil as a graphite cathode and a glass plate with a conductive ITO layer, covered by a CL phosphor as an anode (A); FE image on the anode screen (bright spots), resulted from impingement of electrons at the anode, emitted from the tip of the pencil cathode (B); optical micrograph of the pencil cathode apex (C).

**Figure 2:**
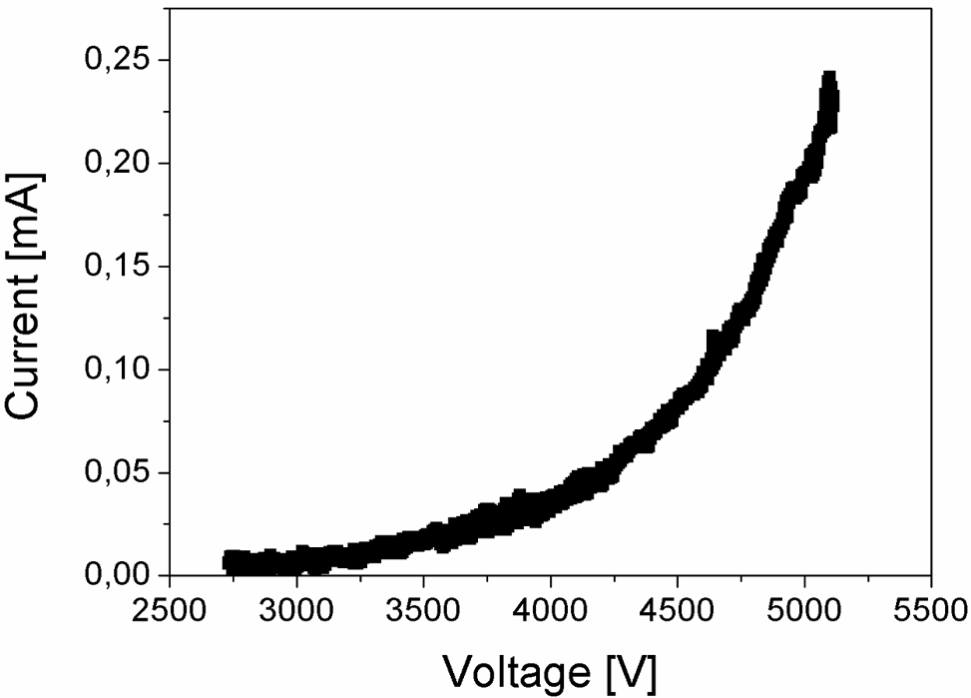
Typical FE current vs voltage dependence measured from the pencil tip.

A simple demonstration of the FE abilities of graphite is presented in the experimental setup shown in Figure 1A, with the cathode made of a usual pencil (see details about the experimental techniques below). The field emission of electrons was observed from the apex of the pencil at a voltage in the range of 2 to 5 kV, applied between the pencil core and the transparent anode. The anode was constructed of a glass plate with a conductive indium tin oxide (ITO) layer and covered by a CL phosphor. The bright spots in Figure 1B indicate the impingement of emitted electrons with the CL anode and demonstrate the presence of a few emission sites on the pencil tip. Figure 1C shows an optical micrograph of the tip. A typical field emission current vs voltage dependence is presented in Figure 2. It demonstrates a rather high density of the obtained electron beam (up to 400 mA/cm^2^) according to our estimation. However, the observed emission was very unstable in time with blinking and moving spots in the FE image and with a variation of the FE current. The averaged total value of the field emission current at constant applied voltage significantly decreased with time within a few minutes. Black traces were observed on the anode screen after these measurements. This demonstrates the process of the deposition of material from the pencil core, degraded under the action of the electric field.

**Figure 3:**
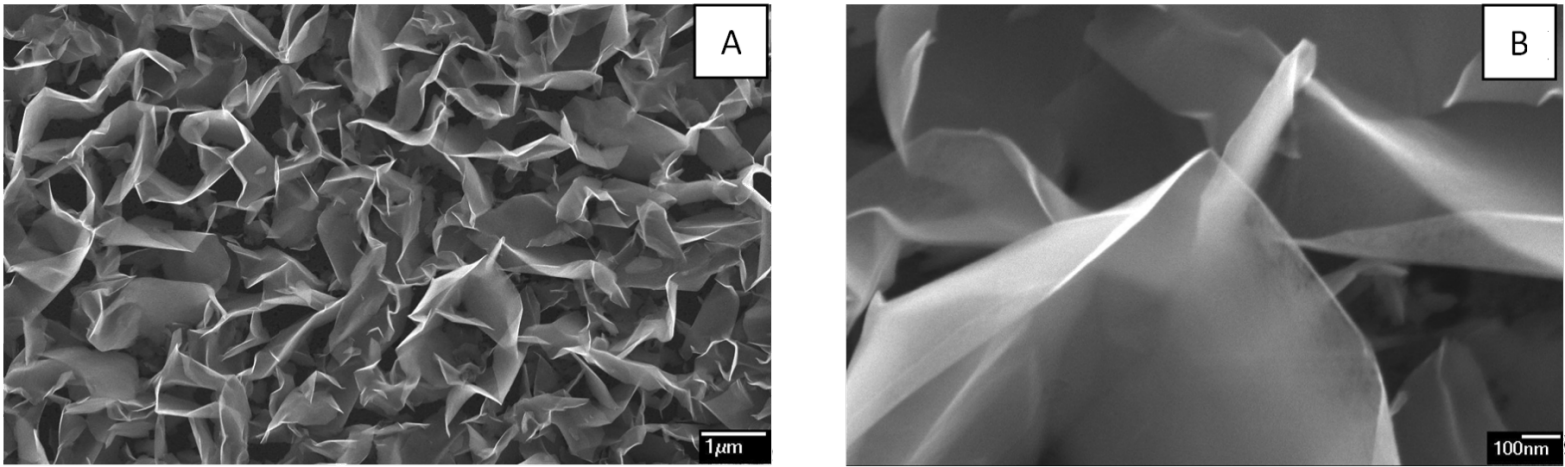
Typical SEM images of NGF material taken with different magnifications: overview of the mesoporous structure of the film (A) and a fragment of a flake transparent for secondary electrons taken with a higher resolution (B).

**Figure 4:**
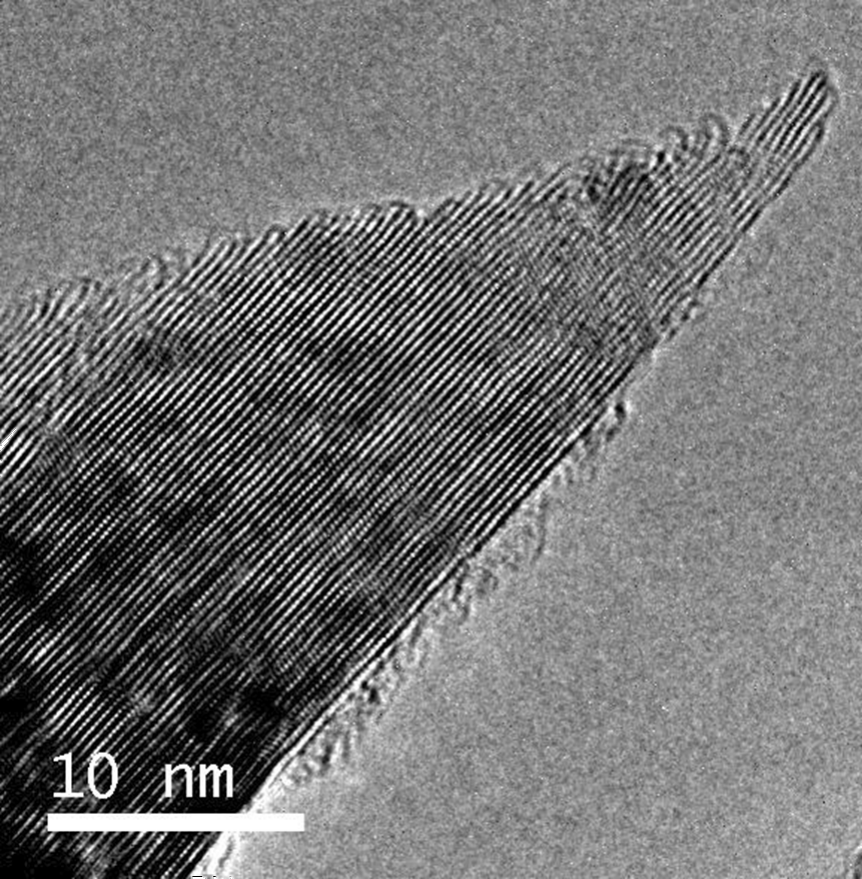
HRTEM image of the top-edge fragment of the graphite flake extracted from NGF material.

These results are quite similar to other materials with a graphitic type of atomic bonding: carbon fibers [9], glassy carbon [10], graphite [11], and carbon nanotubes [12]. The low threshold voltage and the intensive emission properties, which are usually observed for these materials, result from the high aspect ratio of emission sites, which are located on the edges of the graphene monoatomic layers. The low stability of the FE process is stems from the weak interaction between these atomic layers, which lead to their splitting and detachment under the action of the strong electric field. This problem is essentially eliminated in mesoporous nano-graphite film (NGF) material obtained by chemical vapor deposition (CVD) [13–14]. Scanning electron microscopy (SEM) demonstrates that this type of film material consists of tiny graphite flakes (see Figure 3). Transparency of these flakes for secondary electrons in the SEM observations indicates that their thickness is just a few nanometers (see Figure 3B). High resolution imaging with transmission electron microscopy (HRTEM) and electron diffraction analysis [15] confirm this conclusion and indicate that these flakes consist of a few graphene layers (of 5 to 50) oriented predominantly perpendicular to the substrate surface (see Figure 4). The thickness of only a few nanometers results in the high aspect ratio (of 500 to 1000) of these flakes, while the distance between them (in the range of 1 to 4 µm) is close to the estimated optimal value [8].

A significant advantage of this flaky material is its atomic structure at the top edges where adjacent graphene layers are connected with each other. In HRTEM images these connections look like arced structures at the top ends of the graphene layers (see Figure 4). This specific structure results from the material formation process in the plasma activated gaseous environment [13–15]. It is noteworthy that HRTEM images (Figure 4) clearly demonstrate the atomic structure for only a small range of depth in the focal plane. Consequently, we suppose that the pairing for each layer switches from one side to the other, preventing the mutual shift of the atomic layers. This greatly increases the mechanical stability of these flakes. The arced atomic structures formed on the edges of the graphite nanocrystals lead to a local modification of the electronic properties of the material and the formation of a double potential barrier structure. This increases the quantum tunneling probability and the electron field emission intensity [13]. Starting to grow immediately on the Si substrate, these graphite crystallites have an excellent adhesion and electrical contact with the substrate. The mechanical strength and the excellent electrical conductivity at the contact between the nano-graphite flakes and the substrate also provide an improved stability of the NGF cold cathodes. Taking into account that an electron emission from the nano-graphite flakes only occurs from small areas located on their edges, aspect ratios for emission sites are similar to that for CNT emitters. However, cross sections of the flakes are much larger than for nanotubes or nanowires. This circumstance also provides an increased stability of the NGF emitters because of the reduction of the resistivity for individual emitters and the consequent lowering of the Joule heating effect. All together these factors greatly improve the performance of NGF cold cathodes in comparison with other types of cold cathodes. The typical threshold electric field value for NGF cathodes is from 1 to 1.5 V/µm (at emission current density of 1 µA/cm^2^), the emission sites density is up to 10^6^ cm^−2^ (at a current density of about 100 mA/cm^2^), and the maximal emission current is about 1 A/cm^2^ (at applied fields about 2.5 V/µm) [16–17]. The maximal achievable current value requires special attention because of its importance for many practical applications in vacuum electronics.

The local emission current density for an individual emitter is expressed by the Fowler–Nordheim equation [6]

[1]
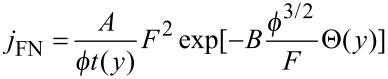


where *j*_FN_ is the current density, *F* is the strength of the local electric field, φ is the work function, *t*(*y*) and Θ(*y*) are tabular functions of *y* = *e*(*eF*)^1/2^/φ; *A* = *e*^3^/(*8πh)*; *B* = 8*π*(2*m*)^1/2^/(3*he*) are constants, *e* is the electron charge, *m* the electron mass, and *h* is the Planck constant. The maximal value of this local current density (*j*_max_) may be estimated by assuming a potential barrier transparency equal to 1:

[2]
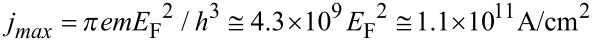


where *E*_F_ = *p*_F_^2^/2*m* 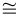 5 eV is the estimated Fermi energy for metals. However, the stable emission current density, experimentally observed at room temperature for a single metallic needle, does not exceed 10^5^ A/cm^2^ [18]. The total value of the emission current density for a multiemitter cathode (*J*) may be estimated as

[3]
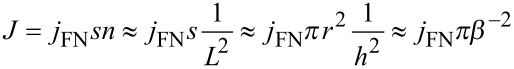


where *s* is the average emitting surface area of the individual emitters, *n* is the density of the emission sites with the geometrical parameters height *h*, radius of emission area *r*, *L* is the distance between two emission sites which is supposed to be in the same order as the height *h*, and β is the so-called field enhancement factor which may be estimated as the aspect ratio *r*/*h*. Thus, the expected range for the averaged emission current density of a multiemitter cold cathode, with aspect ratios of an individual emitter between 100 and 1000, is in the range of 10 to 10^−1^ A/cm^2^ for materials with ‘metallic’ conductivity and work functions about 5 eV. In the particular case of NGF cathodes with aspect ratios of flaky emitters about 500 to 1000, the maximal current density should be about 1 A/cm^2^. This estimation perfectly corresponds to our experimental measurements [19]. A cathode exploitation in an appropriate vacuum environment (on the order of 10^−6^ Torr) with a current value of about 100 mA/cm^2^ provides its long life (more than 10 thousand hours), while current densities higher than 1 A/cm^2^ or a vacuum level reduction lead to its fast degradation within a few minutes [20].

**Figure 5:**
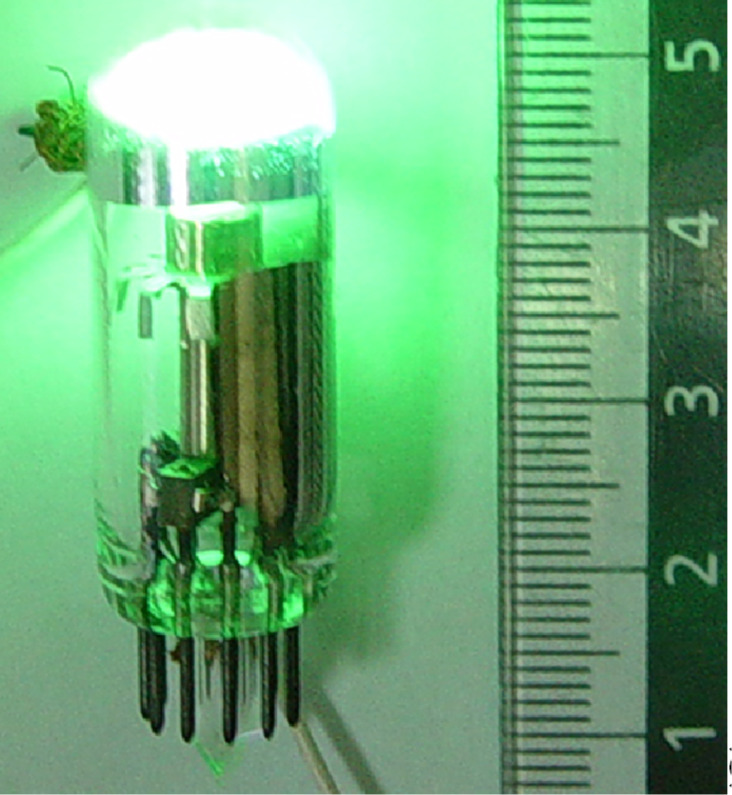
Photograph of the CL diode lamp with an NGF cathode with an emitting surface (of about 1 mm diameter) located at the center of the semispherical surface of the CL anode (of about 5 mm diameter). Applied voltage: pulses of about 15 µs, repetition rate of about 1 kHz with 8 kV amplitude. The image is an adapted version of a previously published graphic in [22] with permission from *J. Nanoelectron. Optoelectron.* © 2009.

Taking into account that the normal operation of a CL phosphor requires current densities in the range of 1 to 10 mA/cm^2^ [3,5], the efficient application of NGF cathodes is possible in CL lamps with a total anode area which is 10 to 100 times larger than the cathode emitting surface. A variant of such a kind of CL lamp has been disclosed in [21] and is presented by a photograph in Figure 5. The FE cathode of this lamp is made of a Ni cylindrical rod (1 mm diameter) with an NGF film covering its end, while the anode is made of an Al thin film deposited over the CL phosphor layer covering the inner semispherical (5 mm diameter) surface of the lamp bulb. The emitting end of the Ni rod is located at the center of the semispherical anode surface and voltage applied between the cathode and the anode is in the range of 5 to 10 kV at a vacuum inside the sealed bulb of about 10^−6^ Torr. Applied with a pulsed voltage (pulse duration of about 15 µs, repetition rate of about 1 kHz) this lamp produces a bright light radiation (see Figure 5).

**Figure 6:**
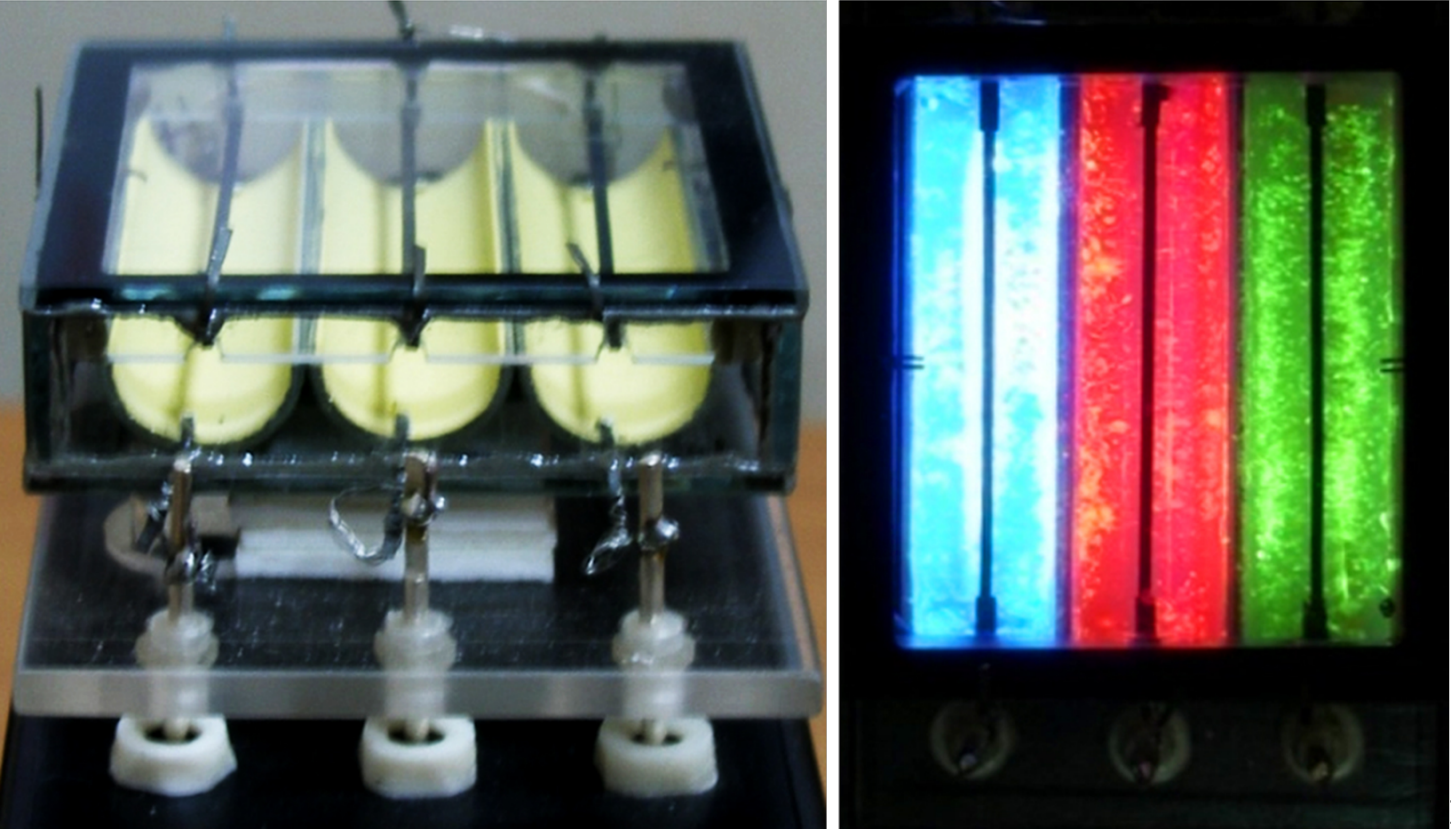
Photographs showing the general design of the CL lamp (left side) and the light emission from RGB segments (right side) with the application of a pulsed voltage (pulse duration 15 µs, repetition rate 1 kHz, amplitude 8 kV) and with a total current per segment of about 0.25 mA. The image is an adapted version of a previously published graphic in [22] with permission from *J. Nanoelectron. Optoelectron.* © 2009.

The light emission of this lamp originates from the cathodoluminescence of the phosphor layer which is located directly under the Al film. This construction is not optimal, similar to other ones reported previously [23–25], because of the energy loss of electrons in the Al film and the loss of light radiation during its propagation through the CL phosphor layer to the output. These disadvantages have been eliminated in a lamp made in accord with the design disclosed in [26]. This type of lamp has a cylindrical diode structure similar to [23] with an anode made of a reflecting Al layer deposited onto half the inner surface of the cylindrical glass bulb and the CL layer deposited over the Al layer. This structure allows direct excitation of the CL phosphor by the electrons emitted from the wire cathode (1 mm Ni wire covered by NGF film) located on the axis of the lamp. The absence of the intermediate material (Al) between the cathode and the CL layer reduces the loss of electron energy and, thus, increases the overall energy efficiency of the lamp. The light generated in the CL phosphor radiates directly from the lamp through the transparent glass surface. An example of such a kind of lamp is presented by the photograph in Figure 6. In this particular prototype three cylindrical diode segments have been placed in one rectangular vacuum envelope. Each segment has its own NGF cathode (40 mm long, 1 mm in diameter) and its own anode (the Al layer is deposited onto inner side of the semicylindrical glass of 8 mm diameter) covered by different phosphor materials to provide red, green and blue colors of radiation.

**Figure 7:**
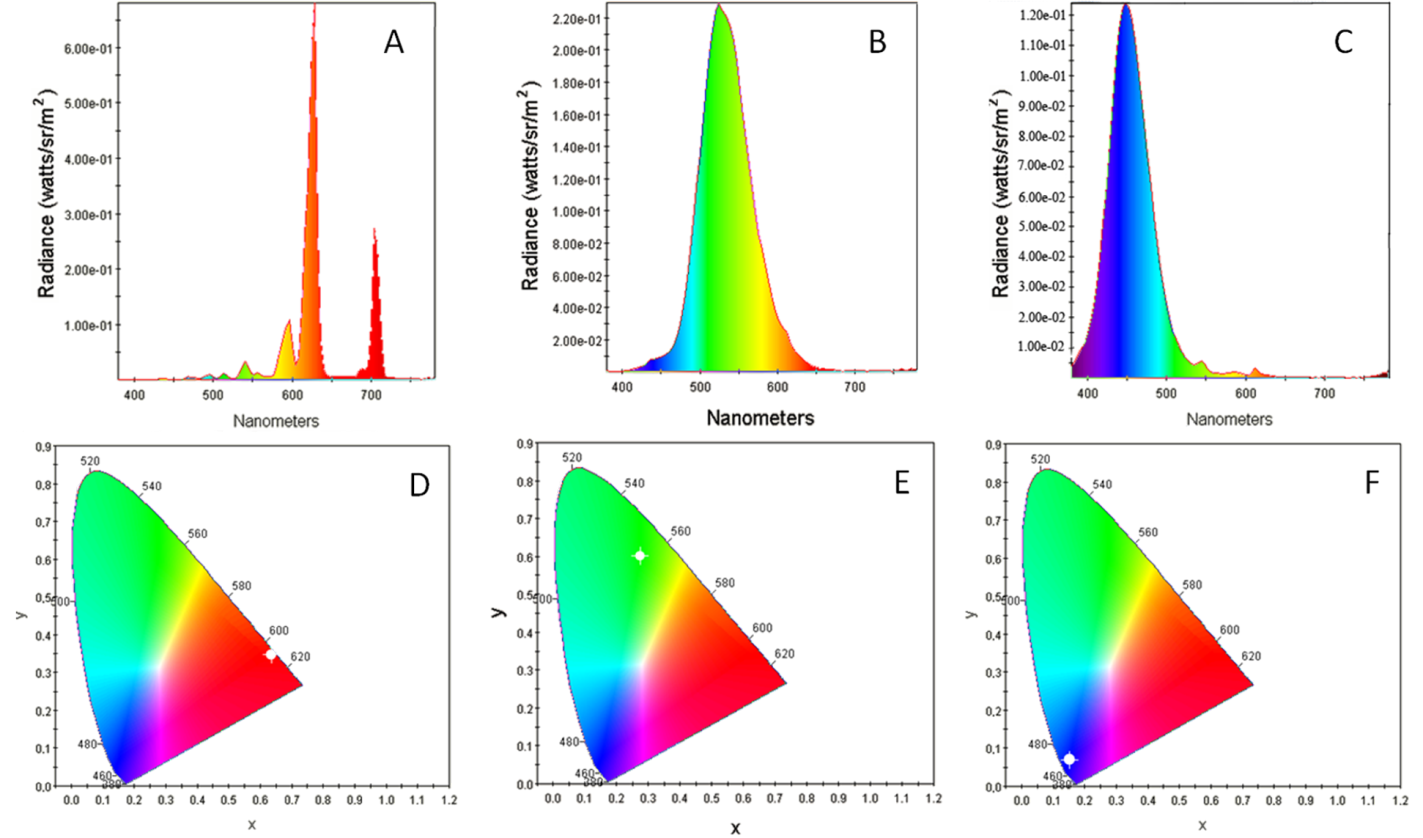
Emission spectra and color coordinates (CIE 1931 Chromaticity Diagrams) for light emitted by red (A, D), green (B, E), and blue (C, F) segments of the CL lamp shown in Figure 6, correspondingly.

The spectral characteristics of the emitted light are determined by the composition of the CL phosphor materials. For the prototype of the CL lamp presented in Figure 6 the following phosphors were used: ZnS CdS:CuAl for green, Y_2_O_2_S:Eu for red, and ZnS:Ag for blue. Measured emission spectra and color coordinates (marked by white circle) for each segment are shown in Figure 7.

With a total power consumption of about 2 W per segment, light intensities correspond to an energy efficiency of about 10% for green light, about 4% for red, and about 3% for blue. These values are the ratios of the total output energy of light and the total input energy of electric current. An inhomogeneity of the light emission over the anode surface (see Figure 6) is owing to the usage of experimental laboratory-level technologies for the cathode and lamp manufacturing, and we believe that it may be eliminated with the application of more advanced industrial methods. This inhomogeneity almost completely disappears with an increase of the input power due to the corresponding increase of radiation brightness. However, the energy efficiency, estimated as the ratio of the energy of the electron beam produced by the NGF cathode to the energy of emitted light, decreases with the rising of consumed power. This is because of the increasing temperature of the phosphor due to the heat generated by the electron bombardment [3]. The use of materials and special designs, allowing more efficient heat dissipation, may reduce the temperature and increase the energy efficiency of the lamps with NGF cold cathodes up to the values predicted from a general consideration of the CL process [3–5]. Standard RGB phosphors used in the present study were designed for an application in cathode-ray tubes (CRT), and their properties were adapted to high accelerating voltages (more than 10 kV) and low currents (1 mA range) of the electron beam. This determines the electrical characteristics of the CL lamp prototypes, including a pulsed operation mode with high voltage short pulses which is necessary to limit the total current. The development of special phosphors, adapted for the application at higher currents and lower voltages, will improve the characteristics of CL lamps and lead to record values of power efficiency.

## Conclusion

Prototypes of CL lamps were manufactured by using cold nano-graphite cathodes. NGF films produced by plasma enhanced CVD consist of a mesoporous graphite flaky material. Each flake is a well ordered graphite crystallite of nanometer thickness, composed of graphene atomic layers, oriented predominantly in the direction perpendicular to the substrate surface. The top edge of the nano-graphite crystallites has a special atomic arrangement providing connections between neighboring graphene layers. This arrangement is responsible for the significant improvement of the mechanical stability of the nano-graphite crystallites as well as for modifications of their electronic properties. Moreover, the special atomic arrangement of the nano-graphite crystallites also results in the formation of a heterogeneous structure with a double potential barrier for the electrons which escape from the graphite to the vacuum under the influence of a strong electric field during the cold emission. The quantum tunneling character of the electron field emission process is responsible for the extremely low energy consumption of the electron beam production. Together with an appropriate lamp design, the cold electron emission from NGF provides an excellent total power efficiency of the CL light source, which is about 10% (for green light) at current laboratory stage. Another advantage of this technology is the potential ability to provide light sources with any colors by simply mixing the phosphor materials.

## Experimental

NGF materials were obtained by chemical vapor deposition (CVD) from a hydrogen/methane gas mixture activated by a direct current discharge. The details of the used home-made CVD system and the corresponding process are described in [13–14]. For the cathode production pieces of Ni wire with 1 mm diameter were placed in a CVD reactor to deposit NGF film material on one of their ends (in case of the lamp presented in Figure 5) or on a cylindrical lateral surface (in case of the lamp presented in Figure 6). The cathodes were used for the lamp production “as grown” without any special post treatments. The as-grown films were inspected with scanning electron microscopy (SEM) by using a Zeiss Leo 1550 instrument and with transmission electron microscopy (TEM) by using a JEOL 3000f instrument.

Field emission tests were performed as described in details in [15–17,19–21] in a vacuum chamber with a basic pressure of about 10^–6^ Torr. The used experimental set-up allows the registration of FE current–voltage dependencies and the distribution of emission sites over the cathode surface in direct current and pulsed regimes corresponding to the device applications. The CL lamp manufacturing was made with the use of standard vacuum electronic technologies, including outgasing with thermal annealing at about 400 °C and porous Ti getter insertion into the sealed device. Commercially available CL phosphor materials with the chemical composition ZnS CdS:CuAl for green lamps, Y_2_O_2_S:Eu for red lamps, and ZnS:Ag for blue lamps were used for the lamp manufacturing. Spectral and luminosity characteristics of CL lamp prototypes were measured by using a HR4000-UV-NIR (Ocean Optics) spectrometer and a LS-110 Luminance Meter (Minolta).
